# Methicillin-Resistant *Staphylococcus aureus* Nasal Colonization among Healthcare Workers at Kampala International University Teaching Hospital, Southwestern Uganda

**DOI:** 10.1155/2019/4157869

**Published:** 2019-03-10

**Authors:** Justus B. Abimana, Charles D. Kato, Joel Bazira

**Affiliations:** ^1^Department of Microbiology and Immunology, Faculty of Biomedical Sciences, Kampala International University, Western Campus, P.O. Box 71, Ishaka, Uganda; ^2^Department of Anatomy, College of Veterinary Medicine Animal Resources & Biosecurity, Makerere University, Box 7062, Kampala, Uganda; ^3^Department of Microbiology, Faculty of Medicine, Mbarara University of Science and Technology, P.O. Box 1410, Mbarara, Uganda

## Abstract

Whereas *Staphylococcus aureus* is a pathogen, it colonizes healthy people as normal flora without causing any symptoms or illness. Probably because of greater exposure, healthcare workers (HCWs) are more colonized, serving as reservoir for endogenous infections as well as dissemination. In developing countries including Uganda, there is scarcity of the literature on *S. aureus* carriage among HCWs, making infection control difficult. This study aimed at determining the nasal carriage rate and comparing the antimicrobial susceptibility profiles of methicillin-resistant *Staphylococcus aureus* (MRSA) and methicillin-susceptible *Staphylococcus aureus* (MSSA) isolates from HCWs of Kampala International University Teaching Hospital. Nasal swab specimens from HCWs were screened for MRSA using both phenotypic and genotypic methods. Antimicrobial susceptibility testing of the MRSA and MSSA isolates was performed using the Kirby–Bauer disc diffusion method. Out of the 97 participants, 28 (28.8%) participants were nasal carriers of *S. aureus* of which 13 (46.4%) were phenotypically MRSA (resistant to cefoxitin) and 8 (28.6%) were genotypically MRSA (had *mecA* gene). Only 6 isolates of the 13 isolates (46%) which showed resistance to cefoxitin had *mecA* gene detectable while 2 (13.3%) of the 15 cefoxitin susceptible isolates were found to carry *mecA* gene. The study thus shows that methicillin resistance in *S. aureus* may not only be determined by *mecA* gene.

## 1. Introduction

*S. aureus* is a very common bacterium that is both a pathogen and normal flora. It can be isolated from many body parts, mostly the nasal cavity and has ability to survive on inanimate objects such as beds, trays, and toilet seats [1, 2]. Approximately 30% of the world human population is persistent carriers of *S. aureus* [3, 4]. The carriage rate is even higher in healthcare workers and clinical students [5]. Factors that determine colonization without showing clinical symptoms are largely unknown [3, 4]; however, variability in host adhesins, immune response, reduced expression of antimicrobial peptides in nasal secretions, polymorphisms in the genes encoding the glucocorticoid receptor, C-reactive proteins, interleukin-4, and complement inhibitor proteins have been associated with persistent nasal carriage [6–9]. Also, studies by Brown et al. demonstrated that after decolonization, persistent carriers often become recolonized with their prior *S. aureus* strain, whereas noncarriers resist experimental colonization [10]. This shows that certain host traits determine colonization.

Colonization of healthcare workers with *S. aureus* is a prerequisite for subsequent endogenous infection and dissemination of the strains to the hospital environment [11]. In 1944, most *Staphylococci* were susceptible to penicillin G; however, due to the misuse of penicillin, many isolates became resistant to the drug by production of *β*-lactamases (penicillinases), coded by *blaZ* gene regulated in an operon manner by a regulatory gene called *BlaR1* [12]. These enzymes degrade the *β*-lactam ring of the antibiotic, making it harmless to the microorganisms [13]. The discovery of *β*-lactamase-resistant penicillins (e.g., nafcillin, oxacilin, cefoxitin, and methicillin) provided a temporary respite [13–15]. However, later methicillin-resistant strains evolved *mecA* gene coding for refractory penicillin binding proteins (PBP2a) which are cell wall-synthesising enzymes that have reduced affinity for penicillins [2]. Currently, MRSA is the most commonly identified antibiotic-resistant pathogen in many parts of the world, both in hospital and community environments [16].

Although the literature is still scarce, MRSA has been reported in different African countries at different prevalences, for instance, 12.7% in Ethiopia [16], 35.8% in Botswana [17], and 46% in Uganda [18]. Colonized healthcare workers have been implicated as major reservoirs of MRSA by different studies [16, 19]. The present study aimed at detecting *S. aureus* and MRSA in HCWs as well as determining antimicrobial susceptibility profile of the isolates.

## 2. Materials and Methods

### 2.1. Study Design

This was a cross-sectional study which involved collection of nasal swab specimens from healthcare workers between September 2016 and July 2017. The participants included nurses, paramedical officers, laboratory technicians, and medical doctors. Nasal swab specimens were collected following previously described procedure [16]. Isolation of *S. aureus* from the samples was done following described bacteriological methods [20]. Antimicrobial susceptibility testing of the isolates was done using the Kirby–Bauer disc diffusion method on Mueller–Hinton agar. Screening for MRSA was done using a cefoxitin disc and PCR amplification of *mecA* gene [21, 22].

### 2.2. Study Area

The samples were collected from Kampala International University Teaching Hospital located in the Ishaka town along Mbarara-Kasese road in Bushenyi District, Southwestern Uganda. Kampala International University Teaching Hospital is sectioned into different departments including Medical, General Surgery, Obstetrics and Gynecology, Pediatrics, Orthopedics, Psychiatry, Dentistry, and Ear Nose and Throat (ENT). The hospital is staffed with about 249 healthcare workers comprising nurses, clinical officers, laboratory technologists, pharmacists, medical officers, and consultants.

### 2.3. Sample Size Determination

The minimum sample size was determined by Slovin's formula stated as *n*=*N*/(1+*N*(*e*)^2^) [23], where *n* = sample size, *N* = population size, and *e* = margin error. In this study, *N* = 249, *e* = 0.08, and the minimum number of participants *n*=249/(1+249(0.05)^2^)=96. This formula was preferred because it is the best formula when the study involves determination of proportion at confidence level 95% and optimal when the proportion is suspected to be close to 0.5.

### 2.4. Isolation and Identification of *S. aureus*

Nasal swab specimens were collected from consenting healthcare workers using a sterile cotton tip swab (Zhejiang Gongdong Medical Technology, China). This was done by rotating sterile cotton swabs in both nares of the participants. The specimens were then transported to Microbiology Laboratory of Kampala International University Teaching Hospital for immediate culture. The specimens were inoculated on 5% blood agar and then incubated at 37°C for 18–24 hours. The colonies showing *β*-hemolysis were subjected to Gram staining, catalase test, mannitol fermentation, and tube coagulase [20, 21]. The isolates that showed positive results for all the above tests were confirmed using Slidex Staph Plus (Biomerieux, France) as *S. aureus*.

### 2.5. Determination of Antimicrobial Susceptibility Pattern

Antibiotic susceptibility testing was carried out using the Kirby–Bauer disc diffusion method on Mueller–Hinton agar (MHA) (Himedia M173-500G, India).

*S. aureus* colonies from mannitol salt agar plates were inoculated in 5 ml of 0.85% saline [24, 25], and the turbidity was adjusted to match 0.5 McFarland standard (1.5 × 10^8^ cfu·ml^−1^). The sterile cotton swabs were dipped into the inoculums and then spread evenly onto MHA. The antibiotic discs including vancomycin 30 *μ*g (Himedia, India), clindamycin 2 *μ*g (Bioanalyse), amoxycillin 30 *μ*g (Oxoid) and levofloxacin 5 *μ*g, (Himedia, India), penicillin G (10 *μ*g), cefoxitin 30 *μ*g, ciprofloxacin 1 *μ*g, ceftazidime 30 *μ*g, amikacin 30 *μ*g, and cotrimoxazole 25 *μ*g (Himedia, India) were applied aseptically to the MHA plates. *S. aureus* ATCC 25923 was used as control strain. The plates were incubated overnight at 37°C, after which the zones of inhibition were measured using a ruler. The interpretation was according to CLSI [25].

### 2.6. DNA Extraction

The DNA was extracted using the boiling method as previously described [21, 22]. Briefly, it involved centrifuging 1 ml of bacterial culture in Luria Bertani (LB) medium at 6800 × g for 3 minutes at room temperature. The pellet was then resuspended in 100 *μ*l of molecular biology grade water and centrifuged at 15000 × g for 10 min. The supernatant was discarded, and the sediment resuspended in 40 *μ*l of molecular biology grade water and boiled at 100°C in a water bath for 10 minutes. This was followed by cooling on ice and centrifuging at 15000 × g for 10 seconds. The supernatant was then used for PCR.

### 2.7. *mecA* Gene PCR Amplification

This was done following the protocol previously described by Elhassan et al. [26]. Briefly, a total volume of 25 *μ*l consisting of 12.5 *μ*l of master mix (containing 2x taq polymerase, dNTPs, and buffer) (Bioline, UK), 0.5 *μ*l of the forward primer, 0.5 *μ*l of the reverse primer, 7.75 *μ*l of PCR water, 1.25 *μ*l MgCl_2_, and 2.5 *μ*l of the template DNA was used for PCR. The 533 bp segment of *mecA* gene was amplified using the primer pair F: 5′-AAAATCGATGGTAAAGGTTGGC-3′ and R: 5′-AGTTCTGCAGTACCGGATTTTGC-3′ (Eurofins Genomics, Germany), as previously reported [27]. The PCR conditions were in accordance with the protocol by Kateete et al. [20] with some modifications. Briefly, it involved initial denaturation at 94°C for 5 minutes followed by 35 cycles of denaturation at 94°C for 30 seconds, annealing at 50°C for 1 minute, extension at 72°C for 1 minute followed by 7 min of final extension at 72°C. The PCR products were resolved by electrophoresis at 125 V for 30 minutes through 2% agarose gel prepared with TAE buffer containing 0.5 mg/ml ethidium bromide [28, 29]. DNA bands on the gel were viewed under the UV digital imaging system. The size of PCR *mecA* amplicons was estimated at 533 bp in comparison with their motilities with those of 50 bp ladder standard.

### 2.8. Quality Control

To avoid false positives, gloves and surgical mask were put on to avoid contamination of the samples. All samples collected were cultured immediately after collection. The reference strain *S. aureus* ATCC 25923 was used as quality control strain during identification and antibiotic susceptibility testing. *S. aureus* ATCC 25923 and *S. aureus* ATCC 43300 were used as negative and positive controls, respectively, during PCR assay.

### 2.9. Data Management and Analysis

Data were entered in Epidata version 4.2 and were analysed using IBM SPSS version 20. The nasal carriage rate of MRSA was calculated as the proportion of individuals positive for MRSA out of the sample population. The chi-square test was used to compare the different groups of healthcare workers. All results with *p* < 0.05 were taken as significant.

### 2.10. Ethical Considerations

The ethical approval was obtained from Institutional Review Board of Mbarara University of Science and Technology (no. 12/09-15). The permission was sought from Directorate of MEDICAL Services of Kampala International University Teaching Hospital. Informed consent was obtained from the participant before starting the study. The identity of the participants was highly concealed.

## 3. Results

### 3.1. Participants' Baseline Characteristics

In total, 97 participants who included doctors, paramedical officers, nurses, and laboratory personnel were involved in the study. Of these, 61 (63%) participants were males and 36 (37%) were females. The participants were stratified according to age and working ward/department. Results are shown in Table 1.

### 3.2. Prevalence of *Staphylococcus aureus*

28.7% (28/97) of the participants were found to be nasal carriers of *S. aureus*. When the nasal carriage rate was compared across sex, no significant difference (*p*=0.458) was observed between males (26.2%) and females (33.3%). The nasal carriage rate significantly increased with age (*p* < 0.01), being highest (75%) in individuals above 35 years (Table 1). Comparisons of nasal carriage rates across different professions did not show any significant differences (*p*=0.225). However, laboratory staff (50%) and doctors (30%) were more colonized. Similarly, the ward/department where the samples were collected did not significantly (*p*=0.433) impact the *S. aureus* positivity (Table 2).

### 3.3. Prevalence of Methicillin-Resistant *Staphylococcus aureus* (MRSA)

#### 3.3.1. Phenotypic MRSA Screening

In order to detect MRSA, cefoxitin discs were used. Among the 28 *S. aureus* isolates, 13 (48%) isolates were confirmed as MRSA. When the positive samples were compared across different age groups, it was shown that age significantly (*p*=0.001) affected MRSA carriage among the study population. However, there were no significant differences observed (*p* values > 0.05) among sexes, professions, and work departments/wards despite different prevalence percentages. The details are shown in Table 3.

#### 3.3.2. Genotypic MRSA Screening

**Figure 1:**
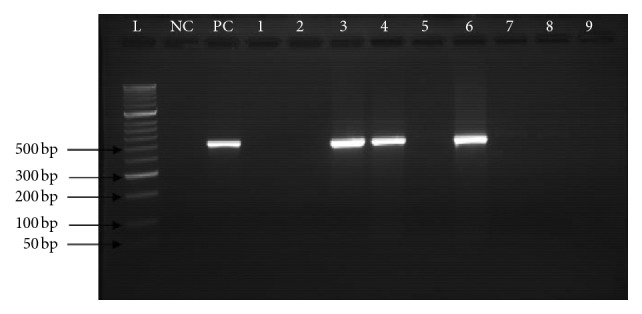
A representative gel showing the amplified product of the 533 bp MecA gene of *S. aureus*. L, ladder; NC, negative control (*S. aureus* ATCC 25923); PC, positive control (ATCC 43300). 3, 4, and 6 are the positive samples; 1, 2, 5, 7, 8, and 9 are the negative samples.

The *S. aureus* isolates were analysed for *mecA* gene using PCR. Among the 28 isolates, 8 (28.6%) isolates had *mecA* gene (Figure 1). Only 6 isolates of the 13 isolates (46%) which showed resistance to cefoxitin had *mecA* gene detectable. On the other hand, 2 (13.3%) isolates of the 15 cefoxitin susceptible isolates were found to carry *mecA* gene. The participants' profession and the ward/department of the work significantly affected the carriage of *mecA* positive strains (*p* < 0.05) while sex and age of the participants did not have any statistically significant effect (*p* > 0.05). The summary of this analysis is presented in Table 4.

#### 3.3.3. Antimicrobial Susceptibility Pattern of MRSA and MSSA Isolates

**Figure 2:**
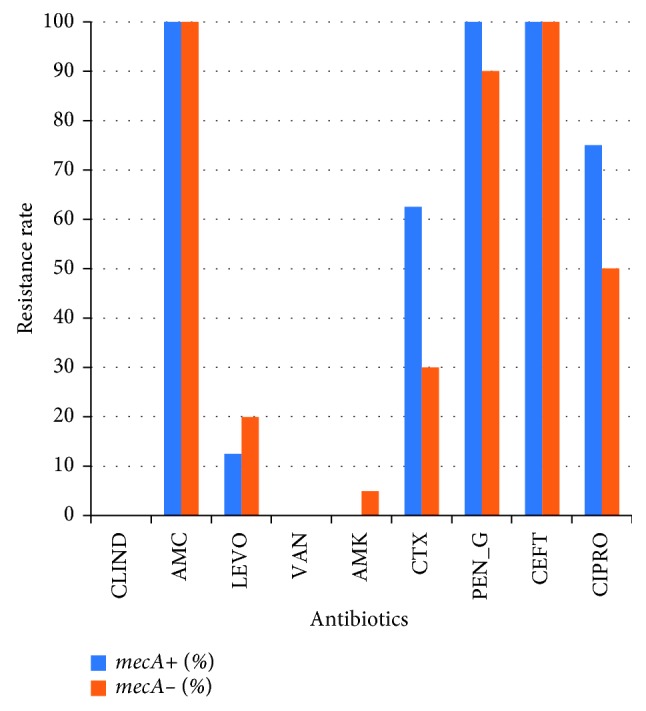
Comparison of resistance rates between *mecA*-positive isolates and *mecA*-negative isolates.

**Figure 3:**
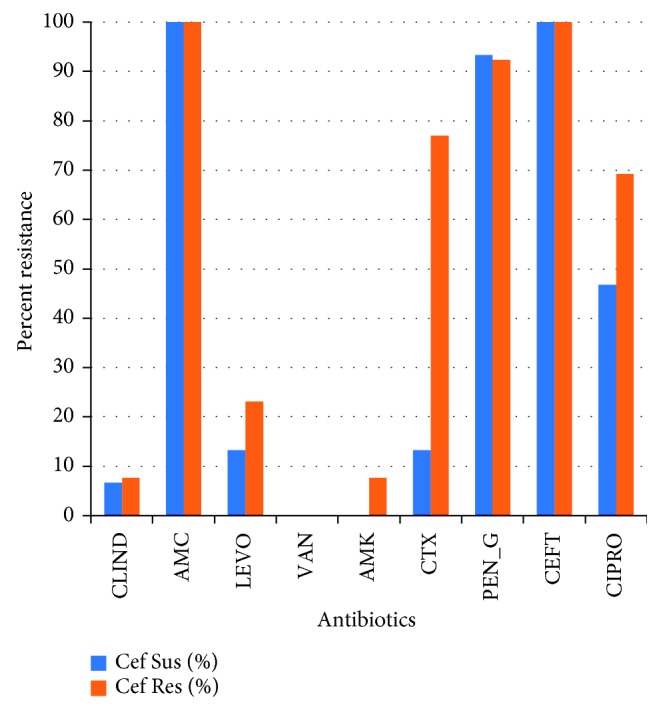
Comparison of resistance rates between cefoxitin-susceptible isolates and cefoxitin-resistant isolates. Cef Sus, cefoxitin susceptible; Cef Res, cefoxitin resistant.

When the isolates were tested for the susceptibility to different antibiotics, MRSA isolates (*mecA* positive) showed a higher resistance rate (Figure 2) than MSSA isolates. Since cefoxitin is used as a predictor of MRSA, the resistance rate among cefoxitin resistant and susceptible isolates was compared. Cefoxitin-resistant isolates showed higher resistance rate to the tested antibiotics than cefoxitin susceptible ones (Figure 3).

*S. aureus* whether MRSA or MSSA isolates showed a high resistance rate to ceftazidime, amoxicillin/clavulanic acid, penicillin G, and cotrimoxazole. On the other hand, the isolates were very susceptible to vancomycin, amikacin, and levofloxacin.

## 4. Discussion

This study determined *S*. *aureus* nasal colonization rate among HCWs, proportion of the phenotypically and genotypically methicillin-resistant isolates and the antimicrobial susceptibility pattern of both MSSA and MRSA isolates.

Results from this study showed that the nasal carriage rate of *S. aureus* among HCWs in Kampala International University Teaching Hospital is 28.8%. Different studies have reported different prevalences, for example, 41.9% in Central Uganda by Kateete et al., 18.3% in Kenya by Omuse et al., 28.8% in Ethiopia by Shibabaw et al., 64% in Nigeria by Akujobi et al., and 31% in Iran and Palestine by Nabil et al. [16, 18, 30–32]. These differences probably are due to differences in the relative abundance of *S*. *aureus* in the respective study sites. Age was associated with *S. aureus* nasal colonization rate. The significance of age in the colonization rate has also been reported by other studies by Hogan et al. and Shibabaw et al. [16, 33]. This may be due to cumulative exposures to the organism which happens with time in hospital setting.

The prevalence of *S. aureus* in this study varied according to professions ranging from 50% among laboratory workers to 22.9% in paramedical officers, although no statistical difference could be observed. This observation agrees with what was reported by Omuse et al. [30]who observed higher prevalence of *S. aureus* nasal carriage among phlebotomists. This suggests that healthcare workers may acquire organisms from patients as they collect laboratory samples. Laboratory staff are also exposed to isolates in the labs, especially if they do not adhere to safety precautions during handling samples/isolates. In this study, the colonization rate among nurses was 25% (3rd highly colonized) as compared to 21.2% in a study in Ethiopia, by Shibabaw et al. [16]. Of the *S. aureus* isolates, 46.4% showed resistance to cefoxitin (hence phenotypic MRSA) and 8 (28.6%) possessed *mecA* gene (hence genotypic MRSA). This may be suggestive that cefoxitin is more sensitive than *mecA* detection even though 3 of the cefoxitin-sensitive isolates carried *mecA* gene. Comparing these findings with other studies, it can be shown that there are slight differences in the prevalence; for example, Zorgani et al. reported 36.8% in Libya [34] while Gebreyesus et al. and Shibabaw et al. reported 14.1% and 44.1% MRSA proportions of *S. aureus* isolated from HCWs in north and northeast Ethiopia, respectively [16, 35]. All these differences in the findings may be due to different geographical distributions of *S. aureus* and relative prevalence of MRSA in different places.

Both the participants' profession and the ward/department where they work statistically affected the genotypic MRSA carriage rate (*p* < 0.05), with only nurses and laboratory staff carrying *mecA*-positive strains. This study concurs with other studies as observed by Shibabaw et al. and Nabil et al. who established nurses to be more MRSA carriers than other professionals [16, 32]. The higher prevalence of genotypic MRSA isolated from lab staff and nurses than other professions could probably be due to frequent exposure to patients.

In the current study, five cefoxitin-resistant isolates were negative for *mecA*. This was also been observed in other studies by Olayinka et al. and Broekema et al. When they performed nitrocefin assay on these isolates, they showed hyperproduction of type A *β*-lactamases [36, 37]. Genome sequencing of another *S. aureus* strain with this trait (called LGA251) found a *mecA* homologue which was 69% identical to *mecA*. To date, it has been called mecC and also confers resistance to methicillin drugs [38]. “Auxiliary genes” identified by Tn551 mutagenesis have also been shown to confer resistance to methicillin drugs in addition to *mecA* gene [39]. This shows that methicillin resistance is complex and changing as new strains are evolving different mechanisms distinct from classical *mecA* gene.

Despite many studies reporting cefoxitin as a surrogate maker for *mecA*, in the current study, 2 (7%) of *S. aureus* isolates were susceptible to cefoxitin but carried *mecA* gene. This trend has been reported by other studies [40, 41]. This could be explained in terms of structural differences in the *mecA* regulatory genes causing low expression [42].

## 5. Conclusions

The present study indicates a high nasal carriage rate of *S. aureus* (28.8%), of which 46% were phenotypically MRSA and 28.6% genotypically MRSA. There is need for more follow-up studies to identify carriers and establish risk factors for colonization in order to put in place decolonization measures. Thirty-eight percent of the *S. aureus* isolates were resistant to cefoxitin but did not carry *mecA* gene while 13% of isolates susceptible to cefoxitin carried *mecA* gene. Therefore, future studies detect not only *mecA* gene when studying genetic basis for methicillin resistance but also other markers such *mecC* gene as well as whole genome sequencing to detect homologues genes that might also cause resistance.

## Figures and Tables

**Table 1:** Participants' baseline characteristics.

Characteristic	Males	Females	Total (%, *n* = 97)	*p* value
Profession				0.01^*∗*^
Doctor	9 (90%)	1 (10%)	10 (10.3%)	
Paramedic	28 (80%)	7 (20%)	35 (36.1%)	
Nurses	14 (38.9%)	22 (61.1%)	36 (37.1%)	
Lab staff	10 (62.5%)	6 (37.5%)	16 (16.5%)	

Age group				0.343
≤25	19 (59.4%)	13 (40.6%)	32 (33%)	
26–30	28 (65%)	15 (35%)	43 (44.3%)	
31–35	7 (50%)	7 (50%)	14 (14.4%)	
≥36	7 (87.5%)	1 (12.5%)	8 (8%)	

Ward/department				0.653
Special clinics	7 (77.8%)	2 (22.2%)	9 (9.3%)	
OPD	4 (50%)	4 (50%)	8 (8.2%)	
Paediatric	12 (67%)	6 (33%)	18 (18.6%)	
Surgical	7 (50%)	7 (50%)	14 (14.4%)	
Obs and Gyne	4 (67%)	2 (33%)	6 (6.2%)	
Laboratory	10 (62.5%)	6 (37.5%)	16 (16.5%)	
Medical ward	7 (87.5%)	1 (12.5%)	8 (8.2%)	
A and E	10 (56%)	8 (44%)	18 (18.6%)	

A and E, accident and emergency; Obs and Gyne, Obstetrics and Gynecology; OPD, outpatients department; ^*∗*^statistically significant.

**Table 2:** Prevalence of *Staphylococcus aureus* nasal carriage.

Characteristics	Number of samples	Positives, *n* (%)	*p* value
Sex			0.458
Male	61	16 (26.2%)	
Female	36	12 (33.3%)	

Age group			<0.01^*∗*^
≤25	32	5 (15.6%)	
26–30	43	12 (27.9%)	
31–35	14	5 (35.7%)	
≥36	08	6 (75.0%)	

Profession			0.225
Doctors	10	3 (30%)	
Paramedics	35	8 (22.9%)	
Nurses	36	9 (25.0%)	
Lab staff	16	8 (50%)	

Department/ward			0.201
Special clinics	9	4 (44.4%)	
OPD	8	3 (37.5%)	
Pediatrics ward	18	4 (22.2%)	
Surgical ward	14	5 (35.6%)	
Obs and Gyne ward	6	1 (16.7%)	
Laboratory	16	8 (50%)	
Medical ward	8	1 (12.5%)	
A and E ward	18	2 (11.1%)	

A and E, accident and emergency; Obs and Gyne, Obstetrics and Gynecology; OPD: outpatients department; ^*∗*^statistically significant.

**Table 3:** *Staphylococcus aureus* resistance to cefoxitin (phenotypic MRSA).

Characteristics	No. of isolates	No. of MRSA	*p* value
Sex			0.404
Male	16	6 (37.5%)	
Female	12	7 (58.3)	

Age group			0.001^*∗*^
≤25	5	1 (20%)	
26–30	12	3 (25%)	
31–35	5	5 (100%)	
≥36	6	4 (66.7%)	

Profession			0.448
Doctor	3	2 (66.7%)	
Paramedic	8	3 (37.5%)	
Nurse	9	5 (55.6%)	
Lab staff	8	3 (37.5%)	

Department/ward			0.420
Special clinics	4	3 (75%)	
OPD	3	1 (33.3%)	
Pediatrics	4	2 (50%)	
Surgical	5	2 (40%)	
Obs and Gyne	1	0	
Laboratory	8	3 (37.5%)	
Medical	1	0	
A and E	2	2 (100%)	

A and E, accident and emergency; Obs and Gyne: Obstetrics and Gynecology; OPD: outpatients department; ^*∗*^statistically significant.

**Table 4:** *Staphylococcus aureus* isolates with *mecA* gene (genotypic MRSA).

Characteristic	Isolates with *mecA*, *n* (%)	*p* value
Sex		0.129
Male	03 (18.8%)	
Female	05 (41.7%)	

Age group		0.244
≤25	1 (20%)	
26–30	4 (33.3%)	
31–35	1 (20%)	
≥36	2 (33.3%)	

Profession		0.002^*∗*^
Doctors	0	
Paramedics	0	
Nurses	03 (33.3%)	
Lab staff	5 (37.5%)	

Department/ward		0.033^*∗*^
Special clinics	0	
OPD	1 (33.3%)	
Pediatrics ward	0	
Surgical ward	1 (20%)	
Obs and Gyne ward	0	
Laboratory	5 (62.5%)	
Medical ward	0	
A and E ward	1 (50%)	

A and E, accident and emergency; Obs and Gyne, Obstetrics and Gynecology; OPD, outpatients department; ^*∗*^statistically significant.

## Data Availability

The tables and figures data used to support the findings of this study are included within the article.
